# Plant Acoustics for Evaluating Vase Life in Chrysanthemums: A Novel and Noninvasive Method

**DOI:** 10.1111/ppl.71036

**Published:** 2026-08-12

**Authors:** Thijs J. Bieling, Satadal Dutta, Berend de Klerk, Malleshaiah SharathKumar, Gerard J. Verbiest

**Affiliations:** ^1^ Department of Precision and Microsystems Engineering Delft University of Technology Delft the Netherlands; ^2^ Plense Technologies B.V Delft the Netherlands; ^3^ Department of Breeding and Research Deliflor Chrysanten B.V Maasdijk the Netherlands

**Keywords:** cut chrysanthemums, cut flowers, hydraulic conductivity, plant hydraulics, postharvest physiology, smart farming, ultrasound pulses, xylem cavitation

## Abstract

Vase life is a key determinant of cut flower quality and market value. Conventional vase life assessment relies on visual inspection and physiological monitoring over several days to weeks, making it labor‐ and time‐intensive. This study introduces a rapid and noninvasive approach using plant acoustics to assess postharvest vase‐life‐related variation in cut chrysanthemum flowers. Six chrysanthemum cultivars were grown under two supplemental lighting treatments (Hybrid and LED) and two planting densities (54 and 74 plants m^−2^). Acoustic monitoring was compared with optical microscopy for the assessment of xylem vessel diameter, while conventional vase‐life testing was performed in parallel. Optical microscopy validated the acoustic measurements, with both methods consistently identifying vessel radii around 10 μm. The acoustic radius ($r_{a}$), derived from pulse settling time measurements, showed cultivar‐ and planting‐density‐specific variation. Linear mixed‐effects modelling demonstrated that the relationship between acoustic radius and vase life differed significantly among cultivars, indicating that a universal relationship across cultivars is not supported. These findings show that acoustic monitoring provides a meaningful noninvasive proxy for vase‐life‐associated stem traits and may serve as a useful cultivar‐calibrated tool for evaluating postharvest longevity in cut chrysanthemums.

## 1 Introduction

Cut flower crops are bred to improve numerous plant traits, including novel color combinations, resistance to stresses (biotic and abiotic), and the longevity of cut flowers, commonly referred to as vase life. Vase life refers to how long a cut flower retains its visual appearance after harvest, which is measured in days (Halevy and Mayak 1979). Improving cut flowers' vase life is essential as it influences product quality, customer satisfaction, and logistical efficiency by reducing wilting risk during transport and handling. Therefore, vase life is crucial in determining product value in domestic and global cut flower markets.

Chrysanthemums are among the top ten globally traded cut flowers. Common issues affecting chrysanthemum cut flowers include leaf yellowing, wilting, and browning of leaves, as well as browning of ray florets or degreening of disc florets (Ferrante et al. 2005; van Geest et al. 2017). Cut chrysanthemums frequently face hydration gaps during harvest, packaging, or cold and dry transport lasting from minutes to weeks. This dry handling method, often used in the cold supply chain for its cost‐effectiveness during large‐scale sea transport, leads to a negative water balance due to continuous transpiration in the absence of water uptake within the closed containers. Additionally, this transport method affects stem xylem hydraulics. The decline in vase life is primarily due to embolism and blockage in xylem vessels postharvest, as xylem hydraulic conductance is crucial for maintaining water balance in the stems (van Ieperen et al. 2002). The formation of embolisms is linked to the specific anatomy and structure of the xylem (Nijsse et al. 2000, 2001).

Stem hydraulic conductivity is largely determined by xylem anatomy, which is influenced by genotype and growth environment interactions. It gradually decreases during vase life, primarily due to air embolism, which begins at the cut surface due to wounding and later spreads throughout the stem, as well as bacterial occlusion. In certain species such as chrysanthemum, Astilbe, and Bouvardia, wound‐induced blockage also contributes to this decline, as a response shaped by phenotype and postharvest environmental conditions (Vaslier and van Doorn 2003; Loubaud and van Doorn 2004). Previous studies have identified a relation between xylem architecture and vase life across various species, including cut roses (Daneshmand et al. 2024), Acacia foliage stems (Damunupola et al. 2011), and the re‐hydration capacity in chrysanthemums (van Ieperen et al. 2002).

The current method of determining the vase life of cut flowers involves monitoring the cut flower transpiration across desiccation by periodically weighing the stems of the flowers placed in vases that contain water and the cessation of weight loss marking the end of vase life (Fanourakis et al. 2021). The current, conventional method is labor‐intensive and often requires several weeks of monitoring. Therefore, there is an increasing demand for alternative and more efficient methods to rapidly assess the vase life of cut flowers.

Given that embolism and blockage are important determinants of vase life, we hypothesize that vase life can be assessed by analyzing the ultrasonic acoustic pulses emitted from plant stems during periods of water stress (greater than −1.8 MPa of tension on the water columns; Tyree et al. 1984) or intense transpiration (Khait et al. 2023). Cavitation‐related processes are a plausible source of these ultrasonic emissions, as the formation and expansion of gas‐filled voids within the xylem can generate transient acoustic signals. However, alternative sources of ultrasonic pulses, including cut‐surface effects, short‐term drying dynamics following hydration, and measurement‐related factors such as sensor coupling, cannot be excluded. Therefore, the recorded acoustic emissions should be interpreted as indicators potentially associated with xylem hydraulic status and structure rather than as direct evidence of cavitation or embolism formation. Recent studies suggest that ultrasonic pulses can be used noninvasively to measure xylem vessel diameter (Dutta et al. 2022), with the vulnerability to embolism being correlated to vessel diameter and characterized by the rate of sound pulse emissions, which can occur up to 40 times per hour (Tyree et al. 1984; Jackson and Grace 1996; Rosner et al. 2012; Nolf et al. 2015).

This study proposes a novel approach for assessing cut flower's vase life through acoustic monitoring. We evaluated the vase life of six chrysanthemum cultivars grown under varying combinations of lighting (high‐pressure sodium, HPS + light emitting diodes, LEDs) and planting densities (54 or 74 m^−2^). Using a conventional method, we assessed the vase life of cut chrysanthemums and analyzed the emitted acoustic pulses to determine the xylem vessel radii. We aim to demonstrate a relation between vase life and xylem vessel radius based on acoustic signals. This study advances the understanding of vase life determination and introduces a novel, noninvasive method for evaluating the postharvest longevity of cut flowers.

## 2 Materials and Methods

### 2.1 Plant Material and Growth Conditions

**FIGURE 1:**
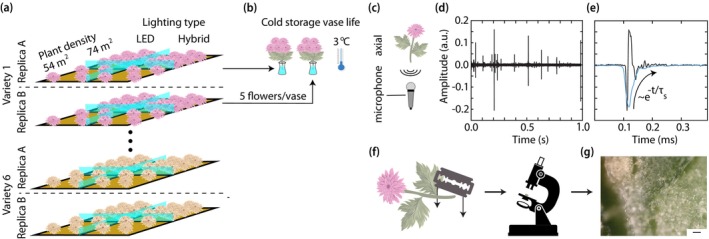
Overview of experimental design and procedures for assessing ultrasound pulses and optical radii of chrysanthemums under varied conditions. (a) Six different cultivars of chrysanthemum were cultivated in a greenhouse under varied lighting conditions (Hybrid/LED) and planting densities (54 and 74 m^−2^). Two replicas (A and B) were grown at different bays within the greenhouse. (b) From each replica and cultivar, five flower stems were selected, placed in a vase, and subjected to vase life testing at room temperature (~20*°*C). (c) Ultrasound emissions from five flower stems were recorded; a typical 1‐s recording is displayed in (d), and a single pulse is illustrated in (e), with an envelope function (red) fitted to estimate the pulse settling time. (f) One flower stem from each vase was selected for further analysis, where its stem was cut into thin cross‐sections for imaging under an optical microscope. (g) A typical microscope image is shown, featuring a scale bar of 50 μm.

Rooted cuttings of six chrysanthemum cultivars (Baltica, Fortune, Midnightsun, Radost, Serenity, Zembla), each with six to seven leaves and an average height of 9–10 cm, were transplanted into a greenhouse at Deliflor Chrysanten in Maasdijk, Netherlands. Two distinct plant densities (54 and 74 plants m^−2^) and two lighting treatments (HPS + LEDs Hybrid and LEDs only) were implemented resulting in four distinct environmental conditions (Figure 1a). The experiment followed a split‐plot‐like design and consisted of two main plots (greenhouse bays) corresponding to the two light treatments (HPS + LED hybrid and LED only). Each bay contained two beds, which served as spatial replicates within the light treatments. Within each bed, the two planting densities (54 and 74 plants m^−2^) were randomly assigned to subplots, and the cultivars were subsequently randomly allocated within each density subplot as sub‐subplots. The experimental unit was defined as a 1.4 m^2^ rectangular sub‐subplot containing a single cultivar at a given planting density under a given light treatment. The higher density of 74 plants m^−2^ represented the standard planting density for commercial production during the summer, while the lower density of 54 plants m^−2^ reflected the standard for winter conditions. Irrigation was applied every 2–3 days, using a chrysant nutrient solution with an electrical conductivity (EC) of 2.7 dS m^−1^ and a pH of 5.6.

The experiment commenced with a two‐week long‐day (LD) photoperiod of 20 h, followed by a short‐day (SD) photoperiod of 13 h. During the LD phase, air temperatures averaged between 20°C and 21°C during the day, while nighttime temperatures were maintained at 18°C. Relative air humidity was kept below 85%, since beyond this threshold stomatal malfunctioning is apparent (Carvalho et al. 2015). Carbon dioxide (CO_2_) supplementation was maintained at 800–1200 ppm (ppm), with set points dynamically adjusted based on ambient growth conditions.

### 2.2 Lighting Conditions

The greenhouse experiment was conducted in bays measuring 30 m × 10 m and included two supplemental lighting treatments (Hybrid and LED) in addition to natural sunlight. One bay received a Hybrid treatment consisting of 12 lamps (six HPS and six LED lamps; Signify), while a second bay received 12 identical LED lamps. The Hybrid lighting spectrum ranged from 400–800 nm, whereas the LED spectrum ranged from 400–700 nm. Light conditions were quantified at canopy level using a spectroradiometer (Specbos 1211, Jeti Technische Instrumente GmbH) at the start of the experiment.

Measured canopy‐level photosynthetic photon flux density (PPFD) averaged 136 ± 5.5 μmol m^−2^ s^−1^ for the Hybrid treatment and 141 ± 6.2 μmol m^−2^ s^−1^ for the LED treatment. The treatments differed in spectral composition, particularly in the red‐to‐far‐red (R) ratio, which was 5.5 ± 0.47 under Hybrid lighting and 13.1 ± 1.9 under LED lighting.

Lighting treatments supplemented natural sunlight and were applied for 20 h day^−1^ during the 15‐day long‐day (LD) phase, resulting in an effective photoperiod of approximately 21 h, and for 12 h day^−1^ during the subsequent short‐day (SD) phase. The estimated contribution of supplemental lighting to total photosynthetically active radiation (PAR) was approximately 40% (±12%) during LD and 45% (±9%) during SD. Based on canopy PPFD, photoperiod, and the estimated contribution of supplemental lighting, the total daily light integral (DLI) was approximately 24.5 mol m^−2^ d^−1^ under the Hybrid treatment and 25.5 mol m^−2^ d^−1^ under the LED treatment during LD, and 13.1 mol m^−2^ d^−1^ (Hybrid) and 13.6 mol m^−2^ d^−1^ (LED) during SD.

Opaque vertical screens were used to minimize light contamination between bays. During the LD phase, screens were partially closed during the day (07:00–16:00) and fully closed at night (16:00–07:00). During the SD phase, bays were completely darkened outside the imposed photoperiod to prevent light contamination from adjacent compartments. Environmental conditions were comparable between treatments. Average air temperature during the LD phase was 20.7°C ± 2.2°C (Hybrid) and 20.3°C ± 2.1°C (LED), and during the SD phase 18.6°C ± 1.6°C (Hybrid) and 18.4°C ± 1.6°C (LED). Relative humidity remained high and similar across treatments, averaging 86.4% ± 6.1% (Hybrid) and 86.2% ± 5.9% (LED). Vapor pressure deficit (VPD) was not explicitly calculated at canopy level and is therefore not reported.

### 2.3 Flower Harvest Conditions and Vase Life Tests

Chrysanthemum plants were harvested when approximately 80% of the flowers were open, which serves as the common commercial harvest‐stage criterion for all cultivars. Although the exact harvest date varied among cultivars due to differences in flowering time and developmental response under short‐day conditions, the same harvest‐stage criterion was applied to ensure comparable developmental maturity at harvest. At harvest, plants were assessed for final leaf number, internode number, branch number, stem thickness, stem length, and plant height. Subsequently, vegetative and floral organs were separated and weighed. These measurements were included to characterize plant developmental status at harvest, although they are not central to the present analysis.

The harvested chrysanthemum stems were obtained for each light treatment (Hybrid and LED) × cultivar (Baltica, Fortune, Midnight Sun, Radost, Serenity, Zembla) × planting density (54 and 74 plants m^−2^) combination from two greenhouse replications, resulting in 20 stems per combination (Figure 1b). The stems were organized into four bundles of five stems, reflecting the replicated greenhouse structure, enclosed in plastic sleeves, and subjected to cold storage at 3°C for 2 weeks to simulate commercial cold‐chain transport conditions.

Following storage, the bundles were divided into two groups: Group 1 for conventional vase‐life measurements and Group 2 for ultrasound pulse measurements as a rapid, noninvasive characterization technique. For conventional vase‐life assessment, stems were removed from the sleeves, trimmed to a uniform length of 60 cm, and placed in glass vases containing distilled water (Ahmadi‐Majd et al. 2021). No antimicrobial agents or preservatives were added in order to evaluate vase life under natural postharvest conditions without external chemical interference. Each vase contained five stems. Because stems within a vase shared the same water environment and could potentially influence one another through solution‐related effects (e.g., microbial growth), they were not treated as independent replicates. Instead, the vase was considered the experimental unit, with two vases per treatment combination corresponding to the greenhouse replications. During the subsequent two‐week monitoring period, vase performance was assessed through regular visual inspections and measurements of relevant quality parameters. Vase life was recorded in days and was considered terminated when leaves began to yellow, when approximately 10% of the leaves exhibited wilting, or when flowers showed petal drop.

### 2.4 Ultrasound Stem Characterization

In addition to the conventional vase life measurements (as described in Section 2.3 on vase life tests), naturally emitted ultrasonic pulses were recorded as a noninvasive approach to characterize the vascular vessel size distribution of chrysanthemum stems (Figure 1c). The measurement protocol was designed to impose a short and controlled dehydration period following stem removal from water in order to elicit ultrasonic emissions associated with xylem tension dynamics. Cut chrysanthemums from Group‐2, stored under cold conditions for 2 weeks, were removed from plastic sleeves, uniformly trimmed to 60 cm, and placed in glass vases filled with distilled water for approximately 10 min hydration. Subsequently, each stem was removed from the vase, surface‐dried, positioned, and recorded over a short time window (~100 s). This protocol was designed to create a standardized transient hydraulic state rather than maintain equilibrium conditions. Ultrasound measurements were performed using an M500‐USB microphone from Pettersson Elektronik AB, with a detection range of 10–250 kHz. The microphone was positioned axially ~1 mm from the cut‐face of the stem, perpendicular to the cross‐section, and recorded ultrasound bursts at a sampling rate of 500 kHz for approximately 100 s at room temperature (20°C). Because of this close‐proximity geometry and sensitivity to alignment and cut‐surface conditions, pulse count and absolute amplitude were not used as primary outputs. Instead, analysis focused on temporal and spectral pulse characteristics, which are expected to reflect intrinsic structural and resonant properties of the stem tissue, consistent with Dutta et al. (2022).

To minimize background noise and spurious signals, a pulse detection threshold of 0.005 arbitrary units (a.u.) was applied at the microphone output. The arbitrary units (a.u.) represent a normalized scale from 0 to 1 corresponding to the dynamic range of the M500‐USB microphone and were used here as an empirical noise‐discrimination threshold rather than a biologically derived cutoff. Figure 1d shows a typical time‐domain signal in which individual ultrasound emissions appear as distinct peaks. For each peak (Figure 1e), the pulse envelope was obtained using the built‐in “envelope()” function in MATLAB, which returns the upper and lower envelopes of the input sequence. The peak of the envelope curve was identified, and the decaying portion was fitted using a least‐squares approach to the exponential function $e^{-t/\tau_{s}}$, where t denotes time, yielding the pulse settling time $\tau_{s}$. We note that parameters such as water potential, mass loss rate, and exact dehydration kinetics were not directly measured and may influence emission dynamics. However, we assumed that intrinsic pulse characteristics remain sufficiently stable over the short recording period (~100 s) to enable comparative analysis across stems; this assumption should be interpreted with caution. Measurements were conducted under controlled laboratory conditions. While ultrasonic detection avoids interference from audible noise, potential contributions from nonbiological ultrasonic disturbances and handling variability remain sources of uncertainty.

### 2.5 Optical Xylem Vessel Diameter

To provide a qualitative comparison with the acoustically inferred vessel dimensions, optical measurements were performed on a limited subsample of stems from Group 2. Specifically, one stem from each bundle of five stems used for ultrasound characterization was selected for optical analysis. The selected stems were sliced into 5 mm sections using a razor blade, as illustrated in Figure 1f. The stem sections were imaged using a Keyence's VHX digital microscope. Although different microscope magnifications were used, all images had a pixel resolution below 1 μm. Xylem vessel diameters (calculated radii) were determined from the images obtained (Figure 1g) by first converting them to a grayscale image and then applying a built‐in “imfindcircles” function in MATLAB with a lower radius threshold of 7 μm and an upper threshold of 100 μm. Using the calculated radii, a histogram was generated, and a log‐normal distribution fit was applied using the least squares method. This approach allowed us to identify the most probable radius, which corresponds to the peak of the log‐normal probability density function.

## 3 Results

### 3.1 Acoustic Radius

**FIGURE 2:**
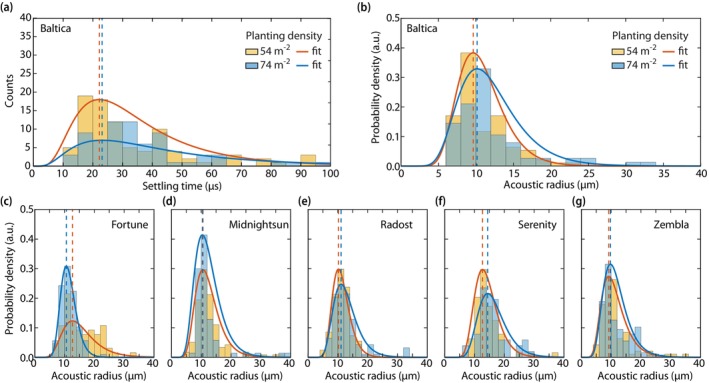
Analysis of acoustic measurements under different planting densities under hybrid lighting conditions. (a) Histogram depicting the distribution of pulse settling times under two planting densities 54 and 74 m^−2^ with the corresponding log‐normal distribution fits (continuous lines). (b) Corresponding histogram of the acoustic radii derived from the pulse settling times. All data were collected from chrysanthemum stems that experienced hybrid lighting conditions and underwent cold storage.

**FIGURE 3:**
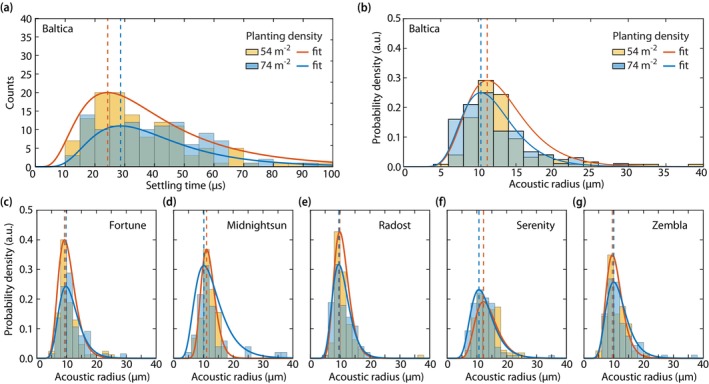
Analysis of acoustic measurements under different planting densities under LED lighting conditions. (a) Histogram depicting the distribution of pulse settling times under two planting densities 54 and 74 m^−2^ with the corresponding log‐normal distribution fits (continuous lines). (b) Corresponding histogram of the acoustic radii derived from the pulse settling times. All data were collected from chrysanthemum stems that experienced LED lighting conditions and underwent cold storage.

To gain insights into the pulse settling time, *τ*_s_ derived from the acoustic pulses under different growing conditions, we categorized the data by each cultivar and condition into respective histograms. Figure 2a illustrates the histograms obtained for the cultivar ‘Baltica’ under hybrid lighting at two different planting densities. By fitting the histogram data with a log‐normal distribution, we found that the most probable pulse settling time for the lower planting density of 54 m^−2^ was 24.2 μs, while for the higher density of 74 m^−2^ it was 28.6 μs. Subsequently, we converted these pulse settling times into acoustic radii (*r*_a_), utilizing the methodology outlined in Dutta et al. 2022:

(1) $$r_{a}=\sqrt{\;\frac{4\;\eta \;\tau_{s}}{\rho }}$$

in which *η* = 8.9 *×* 10^−4^ Pa s and *ρ* = 996 kg m^−3^ are the assumed viscosity and density of the water inside the xylem vessels. The relationship in Equation (1) was derived by modelling the vessel element as a cylindrical acoustic resonator (organ pipe). The results shown in Figure 2b, indicate that for the cultivar Baltica under hybrid lighting conditions, the acoustic radius *r*_a_ is 10.2 μm at a planting density of 54 m^−2^, and 10.7 μm at 74 m^−2^. Further analysis of other cultivars reveals the small differences in *r*_a_ across planting densities (Figure 2c–g). For cultivars Baltica, Radost and Serenity, *r*_a_ is lower at the lower planting density, while for Fortune *r*_a_ is lower at higher planting density. Midnightsun and Zembla cultivars show almost no difference in *r*_a_ between both planting densities. Similar results were observed for the LED lighting conditions as shown in Figure 3. These findings suggest that the relation between *r*_a_ and planting density varies depending on the specific chrysanthemum cultivar, indicating that acoustic measurements may provide cultivar‐specific insights into plant responses under varying cultivation conditions.

### 3.2 Optical Radius

**FIGURE 4:**
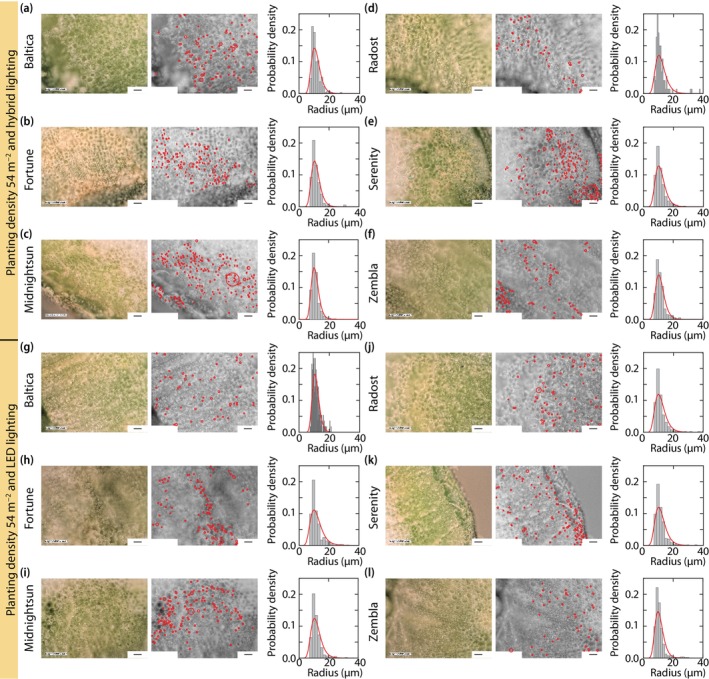
Analysis of optical imaging for chrysanthemum stem cross sections grown at a low planting density of 54 m^2^ under Hybrid and LED lighting conditions. Panels (a) to (i) showcase the analytical process used for optical images of stem cross‐sections from various chrysanthemum cultivars, which were grown under hybrid and LED lighting conditions at a low planting density of 54 m^2^ and subsequently subjected to cold storage. The sequence presented from left to right includes: the original optical image, the converted grayscale image with vascular structures identified in red, and the histogram of vessel radii derived from these images with the corresponding log‐normal distribution fit (red line). The scale bar in the optical images is 50 μm.

**FIGURE 5:**
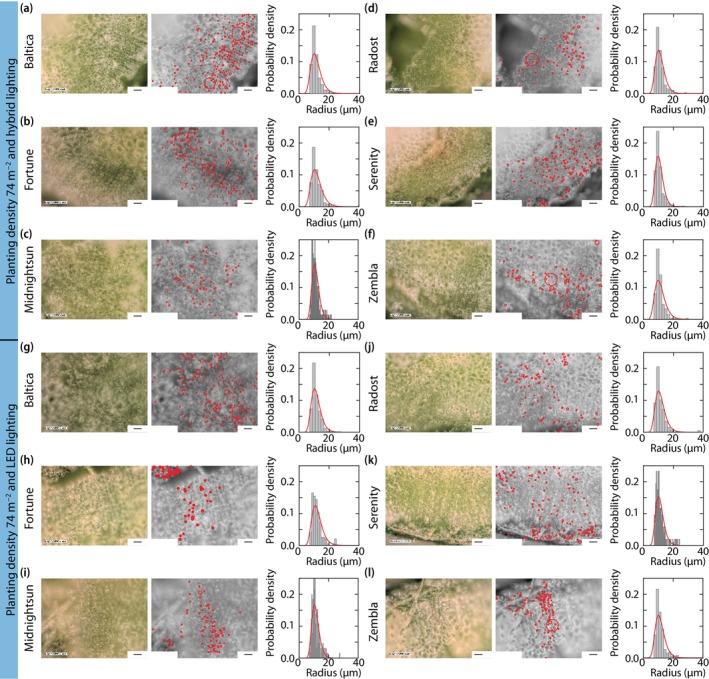
Analysis of optical imaging for chrysanthemum stem cross sections grown at a high planting density of 74 m^2^ under Hybrid and LED lighting conditions. Panels (a) to (i) showcase the analytical process used for optical images of stem cross‐sections from various chrysanthemum cultivars, which were grown under hybrid and LED lighting conditions at a low planting density of 54 m^2^ and subsequently subjected to cold storage. The sequence presented from left to right includes: the original optical image, the converted grayscale image with vascular structures identified in red, and the histogram of vessel radii derived from these images with the corresponding log‐normal distribution fit (red line). The scale bar in the optical images is 50 μm.

To validate the acoustic radii obtained, optical microscopy on flower stems was conducted on stem cross‐sections. For 54 m^−2^ planting density, Figure 4a,l show from left to right: a typical optical image, the analyzed image with red circles indicating the identified vessels, and the histogram of optical vessel radii *r*_o_. The data in the histograms were fitted to a log‐normal distribution (red line) to determine the most probable *r*_o_. A similar figure for 74 m^−2^ planting density is shown in Figure 5. As shown in the histograms in Figures 4 and 5, *r*_o_ approximates 10 μm, demonstrating consistency across different cultivars. These results further confirm the alignment between acoustic and optical measurements, supporting the use of acoustic radii as reliable, noninvasive indicators of xylem vessel size and vase life estimation in chrysanthemum stems.

### 3.3 Relation of Radii With Vase Life

We investigated the relationship between acoustic radius ($r_{a}$) and vase life across chrysanthemum cultivars using linear mixed‐effects modelling to account for the hierarchical experimental design and repeated treatment structure. Three nested models were evaluated, including combinations of acoustic radius, lighting, planting density, cultivar, and cultivar‐specific interaction terms (Table 1). Model 1 does not contain any interaction, whereas Model 2 and 3 include interactions between *r*_a_ and cultivar. Model comparison using likelihood ratio testing demonstrated that inclusion of the interaction between acoustic radius and cultivar significantly improved the model fit compared to Model 1 (p=0.00036), indicating that the relationship between acoustic radius and vase life differs significantly among cultivars. Likelihood ratio testing further showed that removal of lighting treatment did not significantly reduce model performance (LR‐stat = 0.66, *p* = 0.41), and lighting was therefore omitted from the final model.

**TABLE 1:** Comparison of nested linear mixed‐effects models used to evaluate the relationship between vase life and acoustic radius.

Model	Fixed effects included	DoF	Log‐likelihood	AIC	LR‐stat (vs. preceding model)	*p*‐value (vs. preceding model)
1	1 + *r*_a_ + Light + Density + Cultivar	9	−48.83	117.66	—	—
2	1 + *r*_a_ + Light + Density + Cultivar + *r*_a_ × Cultivar	14	−37.40	104.81	22.85	0.00036
3	1 + *r*_a_ + Density + Cultivar + *r*_a_ × Cultivar	13	−37.73	103.47	0.66	0.42

*Note:* Likelihood ratio (LR) testing showed that inclusion of the interaction term between acoustic radius and cultivar significantly improved model fit. Removal of lighting treatment did not significantly reduce model performance, and lighting was therefore omitted from the final model. DoF denotes the number of model degrees of freedom, and AIC denotes the Akaike Information Criterion, where lower values indicate better model fit after accounting for model complexity. LR statistics and associated *p*‐values were calculated by comparing each model with the immediately preceding nested model (i.e., Model 2 vs. Model 1; Model 3 vs. Model 2).

**FIGURE 6:**
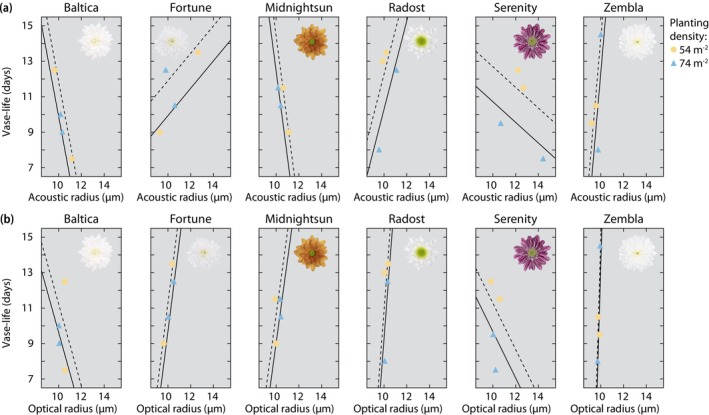
Relationship between vase life and measured acoustic radius ($r_{a}$) (a) and optical radius ($r_{o}$) (b) in chrysanthemum stems subjected to cold storage. Point color indicates cultivar, and marker shape indicates planting density (circles: 54 plants m^−2^; triangles: 74 plants m^−2^). Dashed and continuous lines in panels (a) and (b) indicate cultivar‐specific fitted trends derived from the final linear mixed‐effects model. For each cultivar, dashed and continuous black lines represent the fitted relationships at planting densities of 54 and 74 plants m^−2^, respectively. Optical radius exhibits greater variability and weaker trends across conditions compared to acoustic measurements.

The best‐performing model, Model 3, thus included acoustic radius, planting density, cultivar, and the interaction between *r*_a_ and cultivar. Estimated fixed‐effect coefficients of the final model are shown in Table 2. This analysis indicates that the association between acoustic radius and vase life is cultivar dependent, with both the magnitude and direction of the relationship varying among cultivars. As shown in Figure 6a, vase life ranged between approximately 9 and 14.5 days across all cultivars and growing conditions. While some cultivars exhibited an apparent positive association between $r_{a}$ and vase life, others showed neutral or inverse trends, further supporting the cultivar‐specific nature of this relationship.

**TABLE 2:** Estimated fixed‐effect coefficients of the final linear mixed‐effects model relating vase life to acoustic radius ($r_{a}$), planting density, cultivar, and cultivar‐specific acoustic radius interactions.

Term	Estimate	Standard error	*p*	Confidence interval		
				Lower bound	Upper bound	
Intercept	51.65	11.32	0.0008	26.75	76.56	
Fortune	−42.16	12.05	0.0050	−68.68	−15.64	
Midnight	17.11	22.23	0.4577	−31.81	66.03	
Radost	−59.04	15.35	0.0027	−92.82	−25.26	
Serenity	−27.80	12.36	0.0460	−55.00	−0.59	
Zembla	−106.80	23.67	0.0009	−158.96	−54.64	
Planting density	−0.10	0.03	0.0045	−0.16	−0.04	
*r*_a_	−3.45	1.07	0.0081	−5.80	−1.09	
Fortune × *r*_a_	4.22	1.16	0.0039	1.67	6.77	
Midnight × *r*_a_	−1.46	2.11	0.5046	−6.11	3.19	
Radost × *r*_a_	5.94	1.49	0.0022	2.66	9.23	
Serenity × *r*_a_	2.86	1.16	0.0308	0.32	5.41	
Zembla × *r*_a_	10.92	2.42	0.0009	5.60	16.25	

*Note:* Coefficients are shown with standard errors, *p*‐values, and 95% confidence intervals. Baltica was used as the reference cultivar. Accordingly, the intercept and $r_{a}$ coefficients represent the baseline effects for Baltica. Interaction terms indicate deviations in slope relative to Baltica.

To assess whether similar relationships could be observed using optically measured vessel radii ($r_{o}$), the same linear mixed‐effects modelling approach was applied. In contrast to the acoustic measurements, inclusion of the interaction between optical radius and cultivar did not significantly improve model fit (LR‐stat = 10.96, *p* = 0.052; Table 3), indicating weaker statistical support for a cultivar‐dependent relationship. Consistent with this result, optical radius exhibited greater variability across conditions and less pronounced trends in the data (Figure 6b). These findings suggest that within the present dataset, optical measurements captured weaker cultivar‐dependent relationships with vase life than acoustic measurements. In summary, the findings demonstrate that the acoustic radius provides a meaningful noninvasive proxy for stem traits associated with vase life. However, the cultivar‐dependent nature of the observed relationships indicates that further validation and cultivar‐specific calibration would be required before practical application.

**TABLE 3:** Comparison of nested linear mixed‐effects models used to evaluate the relationship between vase life and optical radius.

Model	Fixed effects included	DoF	Log‐likelihood	AIC	LR‐stat (vs. preceding model)	*p*‐value (vs. preceding model)
1	1 + *r*_o_ + Light + Density + Cultivar	9	−47.24	114.47	—	—
2	1 + *r*_o_ + Light + Density + Cultivar + *r*_o_ ×Cultivar	14	−41.76	113.51	10.96	0.052
3	1 + *r*_o_ + Density + Cultivar + *r*_o_ ×Cultivar	13	−41.84	111.68	0.17	0.68

*Note:* Likelihood ratio (LR) testing showed that inclusion of the interaction term between optical radius and cultivar significantly improved model fit, although the improvement was not statistically significant at *α* = 0.05. Removal of lighting treatment did not significantly reduce model performance and lighting was therefore omitted from the final model. DoF denotes the number of model degrees of freedom, and AIC denotes the Akaike Information Criterion, where lower values indicate better model support after accounting for model complexity. LR statistics and associated *p*‐values were calculated by comparing each model with the immediately preceding nested model (i.e., Model 2 vs. Model 1; Model 3 vs. Model 2).

## 4 Discussion

The results of this study demonstrate a significant association between xylem vessel dimensions inferred from the acoustic method and vase life across chrysanthemum cultivars. The acoustic radius (*r*_a_), derived from pulse‐settling‐time measurements, exhibited cultivar‐ and planting‐density‐dependent variation. Optical microscopy provided qualitative support for the acoustically inferred vessel dimensions, with both methods identifying vessel radii in the range of approximately 10 μm. Mixed‐effects analysis further demonstrated that the relationship between acoustic radius and vase life is cultivar dependent, indicating that acoustic monitoring may provide a useful, novel, and noninvasive tool for cultivar‐calibrated evaluation of postharvest longevity in cut flowers.

### 4.1 Impact of Planting Density on Acoustic Radius and Vase Life

The effect of planting density on acoustic radius was strongly cultivar dependent, with negative, neutral, and positive trends observed among cultivars (Figure 6a; see Section 4.2, variation across cultivars). This highlights the complex, genotype‐specific responses of stem hydraulic properties to planting density, reflecting the interplay between xylem anatomy, plant vigor, and environmental conditions. Despite this variability in acoustic radius, chrysanthemums grown at lower planting densities (54 m^−2^) generally exhibited longer vase life across cultivars (Figures 2, 3, and 6a). One possible explanation is that plants grown at lower densities experience reduced competition for light and nutrients, resulting in greater biomass accumulation, altered hydraulic development, or improved physiological status. Previous studies have suggested that such conditions may promote carbohydrate accumulation and enhance postharvest performance (Fanourakis et al. 2019; Rehling et al. 2021; Vehniwal and Abbey 2019; da Costa et al. 2021; Nawaz et al. 2025). However, biomass production, carbohydrate reserves, and photosynthetic performance were not measured in the present study, and therefore these mechanisms should be regarded as hypotheses rather than direct conclusions from our data.

If differences in carbohydrate reserves were indeed involved, starch could serve as a primary storage carbohydrate, while sucrose may play a key role in translocation and growth. Such reserves could potentially be remobilized to provide energy and adjust osmotic balance, thereby helping maintain cell hydration and supporting water uptake even under stress conditions, including postharvest stress. This may help delay wilting and contribute to extend vase life in plants with greater biomass and higher carbohydrate reserves compared to those with lesser biomass or reserves (Twumasi et al. 2005; Verdonk et al. 2023). Furthermore, maintaining sufficient carbohydrate reserves during postharvest storage may help maintain tissue viability and support continued water transport, thereby contributing to the overall freshness and quality of harvested cut flowers. This observation aligns with the concept that plants with greater biomass and energy reserves can better withstand postharvest stresses, such as water deficits during dry storage conditions in cold chain transport. Additionally, xylem characteristics significantly influence hydraulic conductance, which is key in determining vase life. Planting density may influence xylem development and vessel dimensions, although the direction and magnitude of these responses appear to be strongly cultivar dependent.

In principle, larger vessel diameters can enhance water transport efficiency, which is crucial for maintaining plant freshness and delaying wilting. This increased hydraulic capacity allows the plant to absorb water more effectively during vase storage, keeping the flowers hydrated for extended periods (Olson et al. 2014). The observed association between acoustic radius and vase life (Figure 6a) supports the hypothesis that xylem vessel dimensions contribute to postharvest hydraulic performance, although the direction and magnitude of this relationship depend strongly on cultivar. However, while larger vessel diameters enhance water transport efficiency, they also increase vulnerability to cavitation and embolism, particularly under postharvest conditions where water stress or microbial contamination can occur. Given these trade‐offs, there is likely no universal “optimal” vessel size for maximizing vase life across all species or cultivars. Instead, an intermediate vessel diameter may provide a balance between ensuring sufficient water transport and minimizing the risk of hydraulic failure during vase life. This balance point may also depend on cultivar‐specific traits, such as vessel density, pit membrane properties, and the plant's capacity to prevent or repair embolism. Overall, our results suggest that acoustic radius measurements provide a useful, novel, and noninvasive proxy for stem traits associated with vase life, offering a promising method for evaluating the postharvest longevity of cut flowers, although the underlying physiological mechanisms require further investigation, and additional validation will be necessary before practical application.

### 4.2 Variation Across Cultivars

The relationship between acoustic radius $r_{a}$ and vase life varied significantly across chrysanthemum cultivars (Figure 6a), as confirmed by the significant cultivar interaction term in the mixed‐effects analysis. This indicates that the relationship between acoustic radius and vase life cannot be described by a single universal trend, but instead depends strongly on cultivar‐specific physiological and anatomical characteristics. Some cultivars exhibited positive associations between acoustic radius and vase life, whereas others showed neutral or inverse relationships. This variation is likely due to the genetic characteristics of each cultivar and highlights the complexity of the mechanisms linking xylem structure to postharvest performance.

The cultivar‐dependent nature of these relationships suggests that acoustic monitoring should currently be viewed as a cultivar‐calibrated phenotyping approach rather than a universal predictor of vase life. Consequently, practical deployment in breeding or commercial postharvest applications would likely require cultivar‐specific calibration. Future studies should therefore evaluate the robustness of such calibrations across seasons, production environments, and genetic backgrounds to determine their long‐term applicability.

Although lower planting density tended to enhance both vase life and acoustic radius, the relationship between these parameters is not strictly linear or universal across cultivars. For instance, larger xylem vessels can increase susceptibility to cavitation or embolism, especially under postharvest stress conditions such as dehydration during cold storage. This may explain why cultivars with reduced vase life, despite larger xylem vessels, have lower recuperative potential in the stem after dehydration. While larger vessels facilitate more efficient water transport under ideal conditions, they may also be more vulnerable to failure when exposed to fluctuating hydration levels and dry storage during cold periods (Tyree and Zimmermann 2002). Cavitation, caused by air bubbles forming in the xylem and blocking water transport, can severely reduce vase life (van Meeteren et al. 2006). These findings highlight cultivar‐specific adaptations in xylem structure and function. The observed acoustic radii are shaped not only by growing conditions but also by the cultivar's ability to recover after cold storage. Some cultivars may cope better with the dry storage conditions during the two‐week cold storage period, which can impair water uptake due to air emboli formation or wounding responses. Prolonged darkness during cold storage may also impair stomatal function, further disrupting water balance (van Meeteren et al. 2006; Woltering and Paillart 2018). In addition, cold storage has been reported to reduce chrysanthemum vase life through increased oxidative stress (Fanourakis et al. 2022).

Genetic differences among cultivars may therefore play an important role in determining postharvest performance. For example, cultivars such as “Fortune” may possess traits that allow efficient water transport while maintaining resistance to hydraulic dysfunction, whereas cultivars such as “Baltica” or “Midnight Sun” may be more susceptible to postharvest water stress. These interpretations remain hypothetical, as the underlying physiological mechanisms were not directly measured in the present study. Nevertheless, the observed cultivar‐specific variation suggests that integrating acoustic phenotyping with physiological and genetic information may improve our understanding of the traits governing vase life. Future research should investigate whether genetic markers associated with xylem structure, hydraulic function, and postharvest resilience can be identified and incorporated into breeding programs aimed at developing cut flowers with improved and more predictable vase life.

### 4.3 Validation of Acoustic Radius Using Optical Measurements and Optical Radii Versus Acoustic Radii

We conducted optical microscopy on stem cross‐sections to validate the acoustic radii obtained from ultrasound pulse analysis (Figures 4 and 5). Our measurements of xylem vessel diameters through optical microscopy were consistent with the acoustic radii, confirming that acoustic measurements can be reliably used as a noninvasive alternative to traditional microscopy, as shown in earlier work (Dutta et al. 2022). However, the mixed‐effects modelling between optical measurements and vase life was weaker than that observed with acoustic measurements, particularly in some cultivars (Figure 6b). One explanation for this discrepancy lies in the inherent limitations of optical microscopy. Horizontal cross‐sections of stems may not capture the fine variations in xylem vessel diameter along the full length of the stem that affect water transport efficiency. These longitudinal variations are critical, as they can significantly influence water transport, the formation of air emboli, and ultimately vase life (Nijsse et al. 2001). Furthermore, due to the horizontal cutting of the stems, the photographed vessels often appear oval, making the extracted vessel radius less accurate. In contrast, acoustic measurements are more sensitive to such small variations. Acoustic radii, derived from ultrasound pulses, reflect the functional state of the xylem vessels, capturing data when the entire xylem network is intact and operational. This approach provides a more comprehensive and accurate representation of water transport dynamics than optical microscopy, which offers only a limited snapshot of a specific stem section. For instance, we suspect that the uncertainty in the optical measurements arises from the relatively small vessel sizes compared to the pixel resolution and optical detection limit (~1 μm; Mertz 2019). In contrast, the acoustic radius derived from pulse settling time measurements can be determined with much greater precision. A small but easily detectable change of 1 μs in a pulse settling time of 30 μs results in an acoustic radius change of just 200 nm, which is far below the detection limit of optical methods. This highlights the superior sensitivity of acoustic measurements in capturing minute changes in xylem vessel size. Hence, based on the resonating properties of xylem vessels, acoustic measurements are highly sensitive to minute changes in vessel size and shape. This method offers greater precision, particularly for detecting smaller xylem vessels, which can play a crucial role in water transport. The stronger association between acoustic radius and vase life suggests that acoustic measurements capture key physiological information that optical methods may miss. By providing more accurate data on xylem structure, acoustic measurements present a more reliable approach to evaluating vase life across various cultivars. Therefore, acoustic analysis is valuable for assessing postharvest longevity in ornamental cut flowers.

Future studies should further expand the dataset across additional cultivars and repeated vase‐life experiments to strengthen the statistical robustness of the observed cultivar‐dependent relationships. In addition, refinement of the acoustic measurement protocol and more advanced analysis of acoustic pulse features may further improve the sensitivity and reproducibility of the method. During the present study, acoustic recording times varied between approximately 60 and 120 s. Although the acoustic radius estimates were derived from substantial numbers of recorded pulses and appeared robust within cultivars, future work should aim to standardize measurement duration to further improve comparability across samples and experiments. Future studies should also investigate additional acoustic features, such as resonance peaks described by Dutta et al. (2022), to provide further insight into xylem vessel structure and stem hydraulic properties.

The observed cultivar‐dependent association between acoustic radius (*r*_a_) and vase life demonstrates the potential of acoustic measurements as a noninvasive assessment tool for evaluating postharvest stem characteristics related to cut flower longevity. Such acoustic measurements are particularly valuable for the floriculture industry, where flower quality and longevity are key determinants of the market value. Compared to conventional methods, acoustic measurements offer a rapid and nondestructive means of obtaining information related to xylem structure and function. Together, these findings support the feasibility of acoustic monitoring as a phenotyping approach for cultivar‐specific postharvest quality assessment in chrysanthemum, although further validation will be required before commercial implementation.

### 4.4 Conclusion and Future Perspectives

**FIGURE 7:**
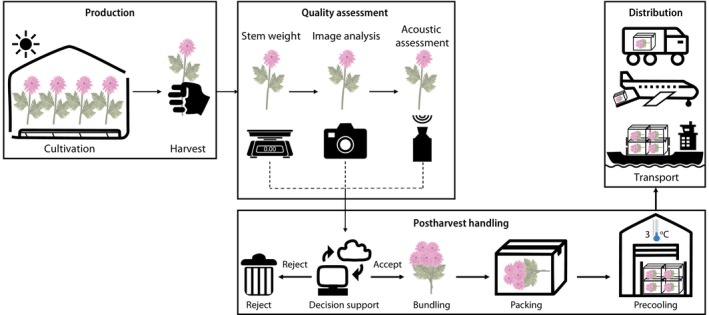
Conceptual illustration of the potential future implementation of acoustic vase‐life estimation within the chrysanthemum postharvest supply chain. Following cultivation and harvest, chrysanthemums are currently sorted and graded using conventional criteria such as stem weight and image‐based quality assessment. Acoustic measurements could potentially be integrated into this workflow to support cultivar‐specific phenotyping, quality screening, and postharvest decision‐making. Accepted flowers would then be bundled, packed, precooled, and transported by truck, aircraft, or ship. The figure represents a prospective implementation pathway and should not be interpreted as a commercially validated system. Further development is required to address measurement throughput, protocol standardization, sensor robustness, calibration stability, and decision‐threshold determination before industrial deployment.

The significant cultivar‐dependent association between vase life and acoustic vessel radii highlights the potential of plant acoustic measurements as a novel, rapid, and noninvasive approach for assessing postharvest stem traits related to cut flower longevity. Our findings indicate that acoustic measurements captured stronger associations with vase life than the optical measurements used in this study, suggesting that they may be more effective in detecting biologically relevant variation in stem hydraulic characteristics. Unlike optical microscopy, which is time‐consuming and invasive, acoustic measurements allow for real‐time, holistic monitoring of cut flower stem hydraulics while the xylem network remains intact. In the longer term, integration of acoustic measurements into cultivation and postharvest supply chains may contribute to improved sorting precision and quality assessment. However, substantial technological development and validation are still required before commercial deployment, particularly with respect to measurement throughput, protocol standardization, sensor robustness, and the establishment of economically relevant decision thresholds (Figure 7). Early identification of flowers with shorter vase life could enable targeted management strategies, such as faster shipping, quicker turnover, or allocation to markets with shorter sales windows. Additionally, acoustic technology could potentially be incorporated into existing quality‐sorting and bunching systems, such as camera‐based machines that assess flower stems based on length, weight, and bud count, provided that challenges related to throughput, standardization, calibration, and industrial robustness can be addressed. Such integration would improve sorting accuracy, automate quality assessments, and reduce reliance on manual inspection. By enabling continuous, data‐driven monitoring, acoustic‐based systems align with the growing demand for smart farming and precision agriculture, supporting optimization of the harvest‐to‐market process while preserving flower quality.

While the present study primarily focused on xylem vessel characteristics and acoustic measurements to assess the water uptake component, we recognize that water loss primarily through transpiration was not directly evaluated. Given that vase life is inherently governed by the dynamic balance between water uptake and water loss, this represents a limitation of the current work. However, we view this as an opportunity to advance the research further. Building on the findings presented here, our ongoing investigations aim to integrate water loss measurements with xylem anatomical assessments and acoustic monitoring. Through this approach, we seek to establish a more comprehensive understanding of postharvest water relations and to explore the potential of acoustic emissions as a noninvasive, labor‐efficient proxy for both water uptake and water‐loss dynamics. We consider this study an important first step in that direction, providing a foundation for future refinement of acoustic‐based tools for postharvest evaluation of cut flowers.

Furthermore, to fully unlock the potential of acoustic monitoring in postharvest quality assessment, further research should prioritize the development of standardized protocols for acoustic measurements, including optimal microphone positioning along the stem to account for the longitudinal distribution of xylem vessels. Enhancing sampling rates and refining methodologies to ensure reproducibility and accuracy across different cut flower species and growing conditions is essential. Given the variation observed across cultivars, further investigation into cultivar‐specific acoustic–vase‐life relationships is needed to better understand genotypic differences in xylem architecture and their influence on postharvest performance. At present, the technology should be viewed primarily as a proof‐of‐concept, cultivar‐calibrated phenotyping and diagnostic approach rather than a fully mature commercial sorting technology. Future work should therefore focus on independent validation across seasons and production environments, assessment of calibration stability, and evaluation of industrial deployment requirements. Additionally, extending acoustic monitoring to other cut flower crops where water transport significantly impacts postharvest quality could broaden the application of acoustic techniques. Integrating acoustic data with environmental variables such as humidity and temperature could further support the development of future predictive models of plant health and postharvest longevity. Pursuing these research directions could unlock the full potential of acoustic monitoring in plant science.

## Author Contributions

T.J.B., S.D., B.d.K., and G.J.V. performed the ultrasound recordings. T.J.B. performed the optical microscopy measurements and MSK performed the vase‐life tests. T.J.B., S.D., and G.J.V. performed the data analysis. S.D., M.S.K., and G.J.V. conceptualized the experiments and supervised the project. All authors were involved in drafting and proofreading the manuscript.

## Funding

This work was supported by 4TU Plantenna and Thematische Technology Transfer Voucher.

## Disclosure

The AI tool was not used to generate scientific content, perform data analyzes, interpret results, or draw scientific conclusions. All scientific content was critically reviewed, revised where appropriate, and approved by the authors, who take full responsibility for the final manuscript.

## Conflicts of Interest

Thijs J. Bieling and Berend de Klerk were the cofounders of the former startup Plense Technologies B.V. Gerard J. Verbiest served as a technology advisor for Plense Technologies B.V. The other authors declare no conflicts of interest.

## Data Availability

The data that support the findings of this study are available on request from the corresponding author. The data are not publicly available due to privacy or ethical restrictions.
